# Supplementary material for: Discrete and conserved inflammatory signatures drive thrombosis in different organs after Salmonella infection
Source: Nat Commun. 2025 Mar 10;16:2356. doi: 10.1038/s41467-025-57466-6 (PMC11894133; doi:10.1038/s41467-025-57466-6)
Supplement: Supplementary file 1 — Supplementary Information [file 41467_2025_57466_MOESM1_ESM.pdf]

Discrete and conserved inflammatory signatures drive thrombosis in different organs after *Salmonella* infection

Marisol Perez-Toledo, Nonantzin Beristain-Covarrubias, Jamie Pillaye, Ruby R Persaud, Edith Marcial-Juarez, Sian E. Jossi, Jessica R. Hitchcock, Areej Alshayea, William M. Channell, Niek TJ Wiersma, Rachel E Lamerton, Dean P Kavanagh, Agostina Carestia, William G Horsnell, Ian R. Henderson, Nigel Mackman, Andrew R Clark, Craig N Jenne, Julie Rayes, Steve P. Watson, Adam F. Cunningham[1]*

***Correspondence:** Adam F. Cunningham and Steve P. Watson
email: a.f.cunningham@bham.ac.uk; s.p.watson@bham.ac.uk

Supplementary figures and information

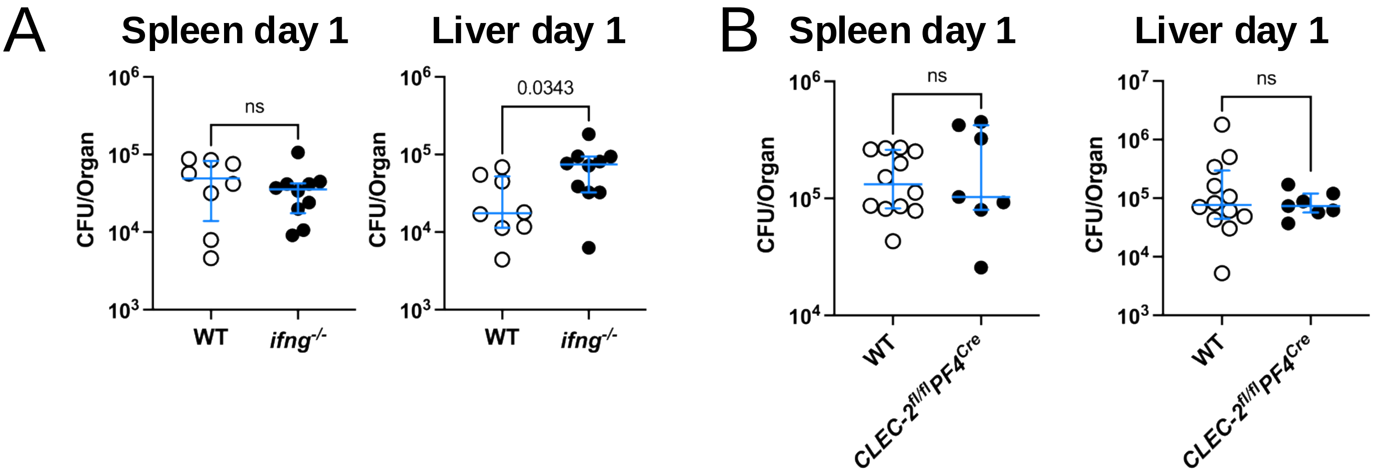

Supplementary figure 1. **Colony Forming Units (CFU) in spleen and liver of mice infected for 24 h with 5x10^5 CFU STm SL3261.** (A) CFU in spleens (left) and livers (right) 1-day post-STm infection, in WT (n=8) and IFN-γ-deficient mice (n=10). Each point represents data from a single mouse. The data within the figure was generated from combining the results from two independent experiments. (B) CFU in spleen (left) and liver (right) 1-day post-infection, inn WT (n=12) or CLEC-2$^{fl/fl}$PF4^{Cre} (n=7) mice. Each point represents data from a single mouse. The data within the figure was generated from combining the results from two independent experiments. Horizontal lines depict the median. Error bars depict the 75th-25th interquartile range (IQR). Statistical analyses were performed using the Mann-Whitney test. ns=non-significant.

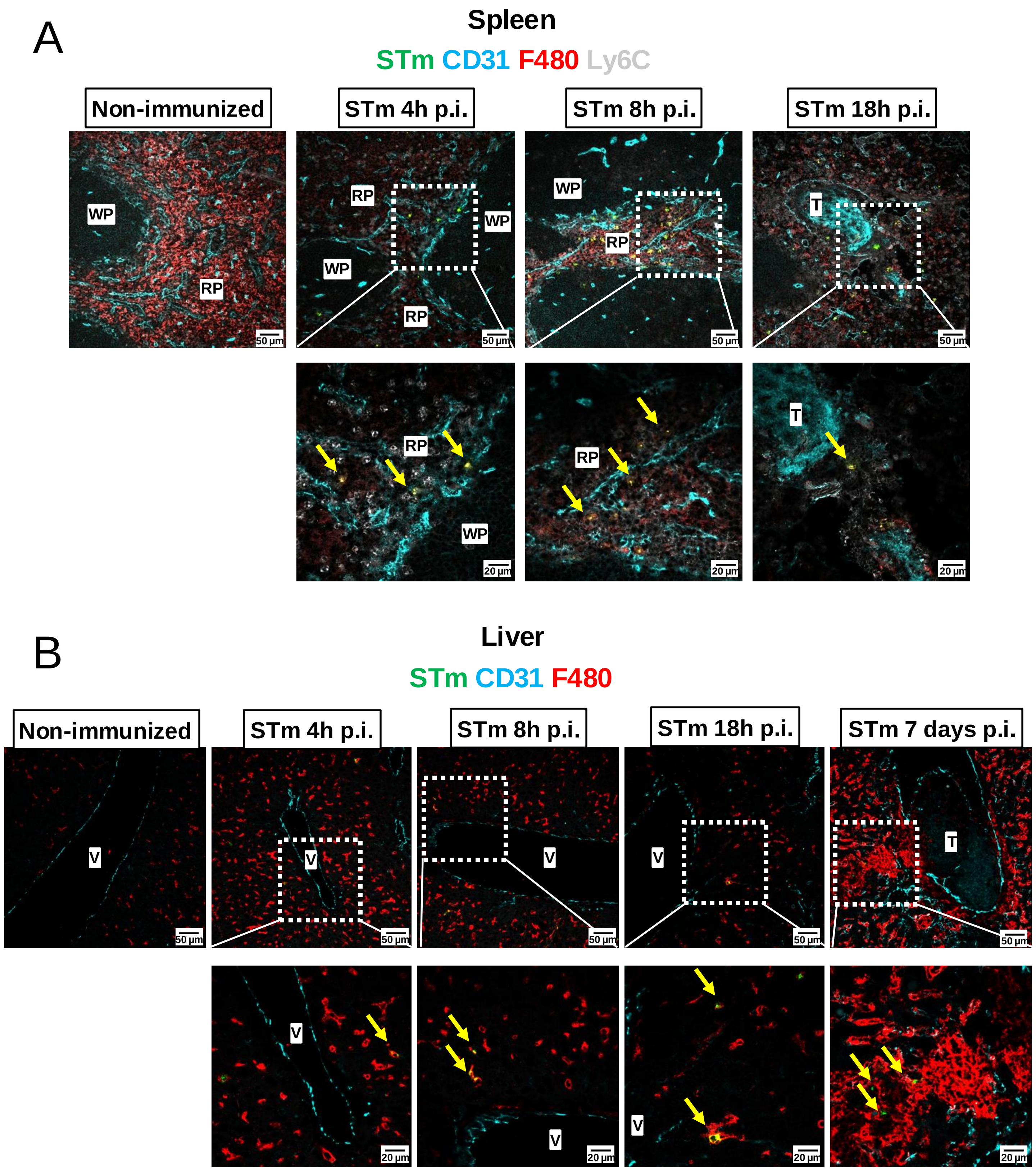

Supplementary figure 2. **Distribution of STm within spleens and livers after infection**. C57BL/6 mice were infected with 5x10^5 CFU STm SL3261 i.p. for 4, 8, 18 hours or 7 days. (A) Representative immunofluorescence of spleen sections stained for STm (green), CD31 (blue, F4/80 (red) and Ly6C (grey) at the indicated time points. (B) Representative images of liver sections stained for STm (green), CD31(blue) and F4/80 (red). The bottom panel is a higher magnification image of the upper panel marked with a dotted box. Yellow arrows indicate the location of bacteria. Groups contain 4 mice per group. RP=Red pulp, WP= white pulp, T=thrombi, V=Vessel.

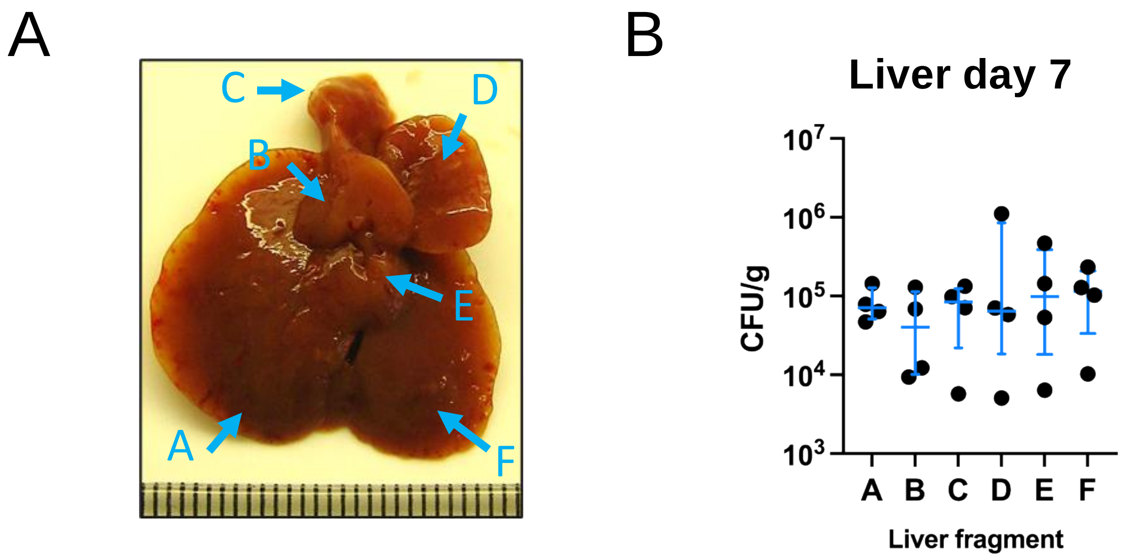

Supplementary figure 3. STm is distributed to similar densities throughout the liver after i.p. infection. (A) C57BL/6 mice were infected with $5x10^5$ CFU STm SL3261 i.p. for 7 days. Livers were dissected and divided into 6 fragments (areas A-F), as depicted in (A). (B) Colony Forming Units (CFU) per gram of liver in fragments A-F. Bacterial culture was measured in each area as described in the methods section. Each point represents data from a single mouse. Horizontal lines depict the median. Error bars depict the 75th-25th interquartile range (IQR). Statistical analyses were performed using the Mann-Whitney test. ns=non-significant

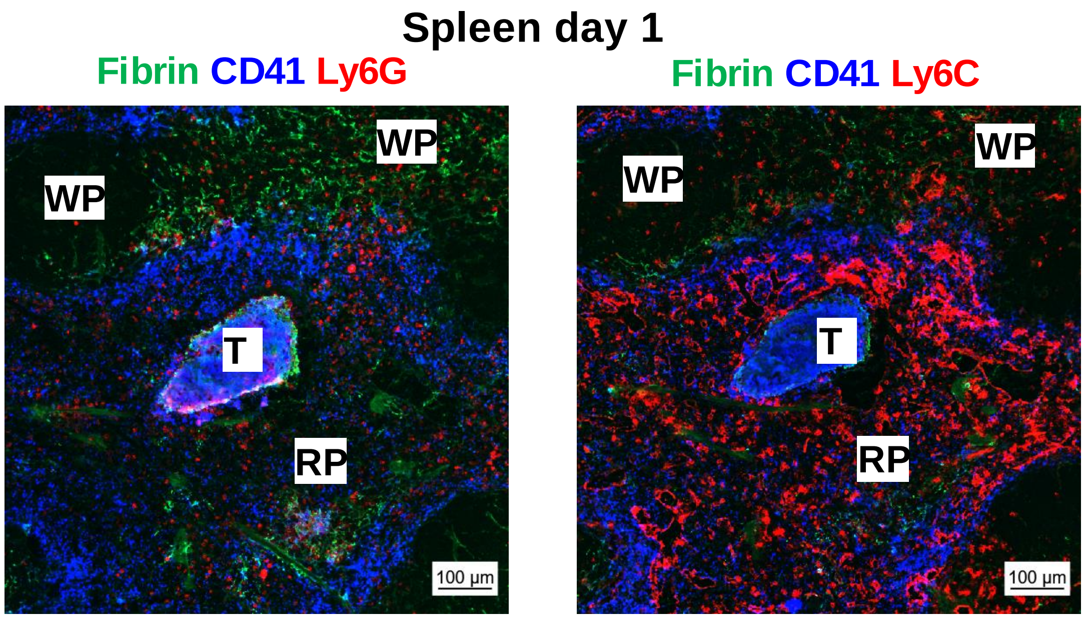

Supplementary figure 4. LyC+ monocytes and neutrophils are primarily localized within the red pulp of the spleen 1 day after infection with STm. C57BL/6 mice were infected with $5x10^5$ CFU STm SL3261 i.p. for 1 day. Serial spleen tissue sections were stained for fibrin (green), CD41 (blue), Ly6G (red, left panel) or Ly6C (red, right panel). Representative staining of 8 mice. RP = red pulp, WP = white pulp, T = Thrombi.

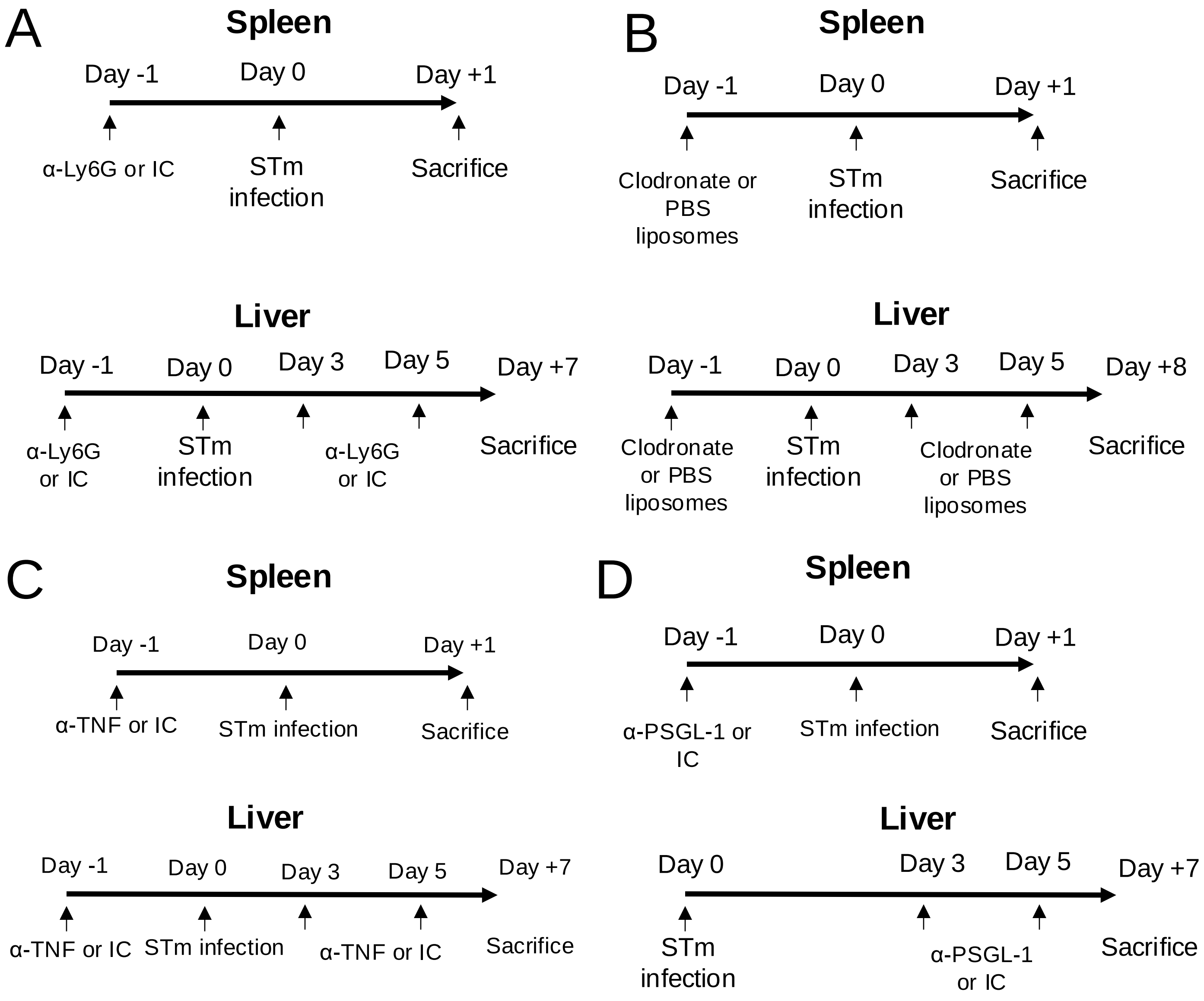

Supplementary figure 5. **Experimental plans used to modulate the host immune system using antibodies or clodronate liposomes.** (A) monocytic lineage cells (B) blocking TNF (C) or blocking PSGL-1 (D). Briefly, 6-8 weeks old C57Bl/6 mice were infected with $5x10^5$ CFU i.p. with STm SL3261. Antibodies or controls were administered via i.p. in the time points indicated and as described in the methods section. Substance concentrations are found in Supplementary Table I.

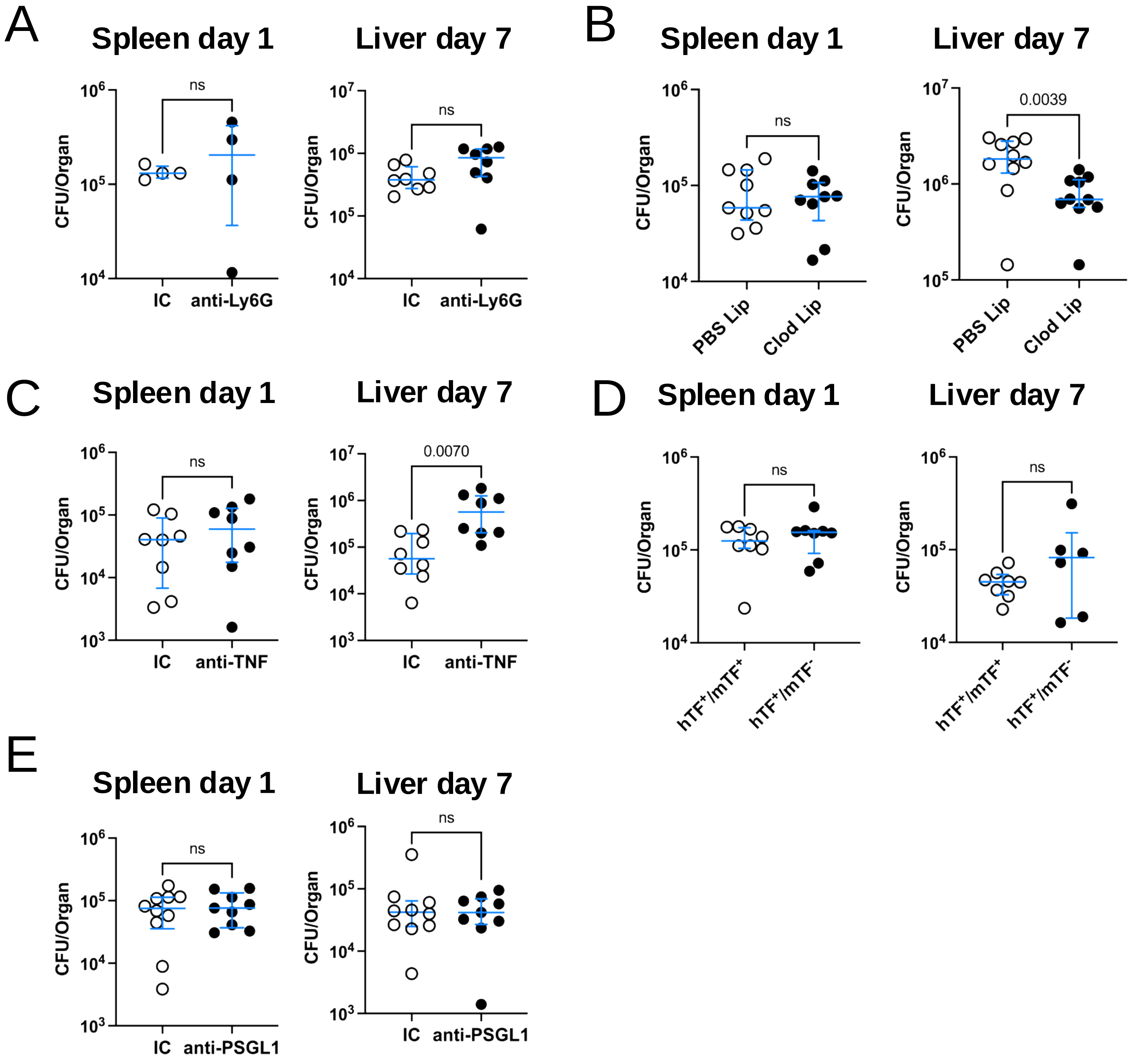

Supplementary figure 6. **Colony Forming Units (CFU) recovered from the spleens or livers of mice after infection.** Mice were infected with $5x10^5$ CFU STm SL3261 and bacterial numbers assessed at day 1 post-infection for spleens and day 7 post-infection for livers. (A) isotype control or anti-Ly6G treated mice, (B) PBS liposomes or clodronate liposomes treated mice, (C) Isotype control or anti-TNF treated mice, (D) isotype control or anti-PSGL-1 mice and (E) TF-sufficient controls (hTF^+/mTF^+) or low TF mice (hTF^+/mTF^-). Each dot represents an individual mouse. The data within the figure was generated from combining the results from two independent experiments. Horizontal lines depict the median. Error bars depict the 75th-25th interquartile range (IQR). Statistical analyses were performed using the Mann-Whitney test. ns=non-significant.

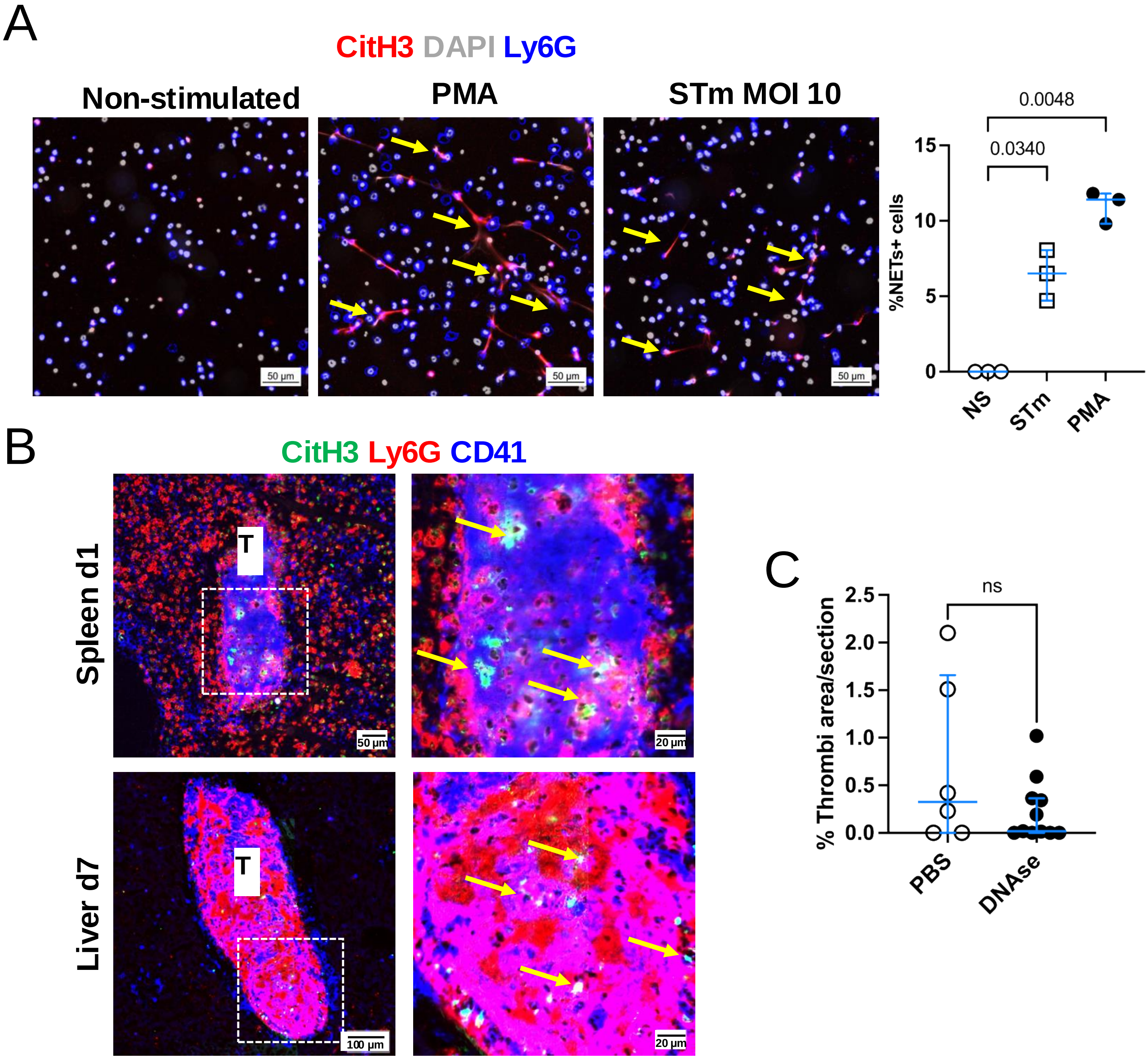

Supplementary figure 7. In vitro and in vivo infection with STm induces NETs-associated responses. (A) Neutrophils purified from the bone marrows of C57BL/6 mice were stimulated with phorbol 12-myristate-13 acetate (PMA) or infected with STm SL1344 for 4 hours (Multiplicity Of Infection (MOI) =10) and stained for citrullinated histone 3 (CitH3, red), DAPI (grey), and Ly6G (blue). Yellow arrows identify NETs. The graph shows the frequency of NETs+ cells (defined as dual CitH3+ and elongated DNA staining). Each point represents an independent experiment. Statistical analyses were performed using the one-way ANOVA with Dunnett's multiple comparisons test. (B) Representative images of a splenic or liver thrombus (1 and 7 days post-infection respectively) stained for citrullinated Histone 3 (CitH3, green), Ly6G (Red), and CD41 (blue). The right panel shows a higher magnification image of the area in the white dotted box. Yellow arrows label examples of CitH3+ staining. (C) C57BL/6 mice were infected with $5x10^5$ CFU STm SL3261 i.p for 6 hours before receiving 60 µg of DNAse I (n=11) or PBS (n=6) i.v.. All mice were culled at 24 hours post-infection. Thrombi were quantified in spleens sections as described in the methods. The graph shows the frequency of section area occupied by thrombi. Each point represents data from a single mouse, and the data in the graph is generated from combining results from two independent experiments. Error bars depict the 75th-25th interquartile range (IQR). Statistical analyses were performed using the Mann-Whitney test. ns= non-significant. T=Thrombus.

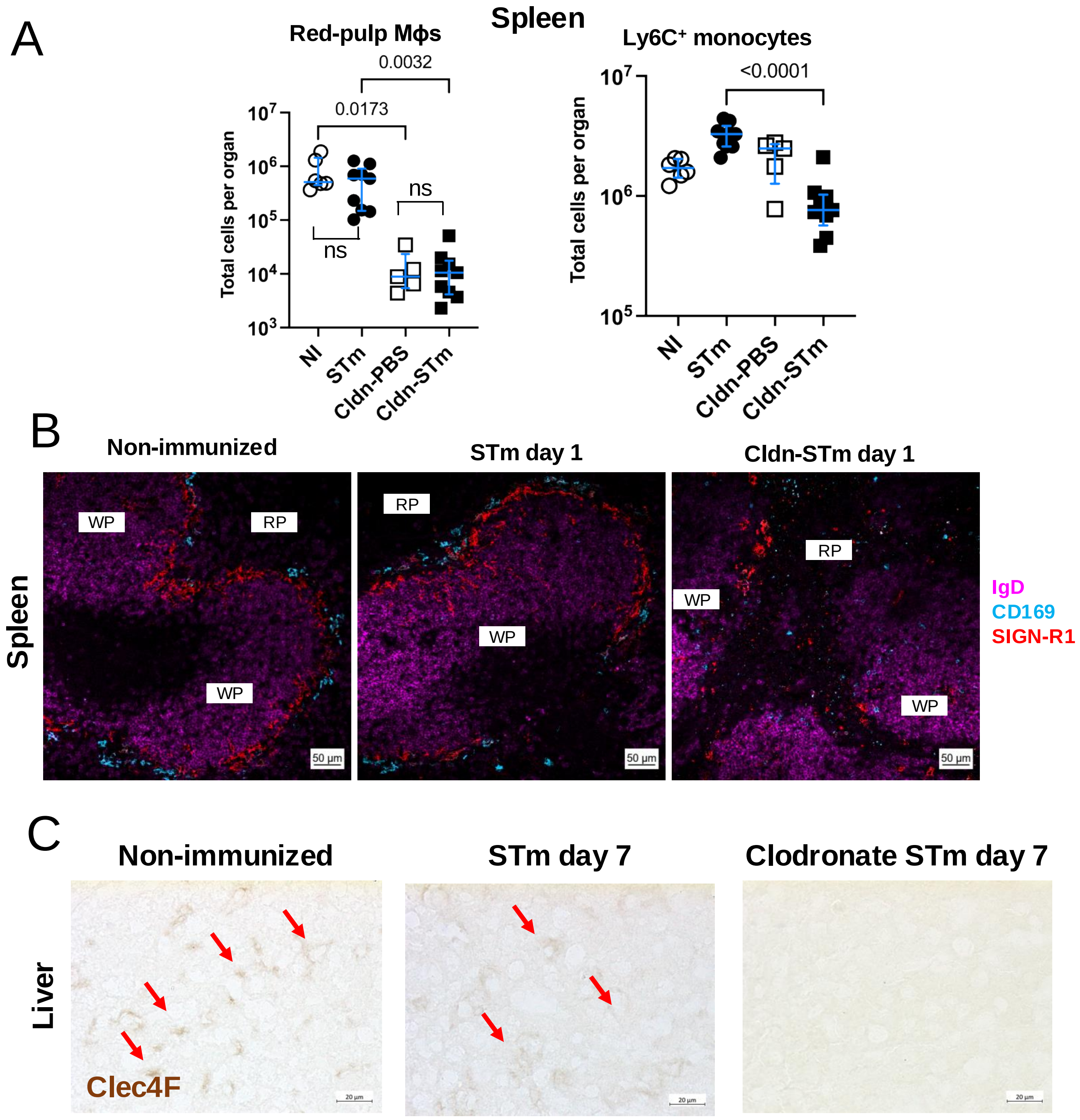

Supplementary figure 8. Effect of clodronate liposomes on monocytic-lineage cells in the spleen and liver. C57BL/6 mice were injected i.p. with clodronate (cldn, n=9) or PBS liposomes (PBS, n=5) 24 hours before i.p.infection with 5x10^5 CFU STm SL3261. Non-infected mice (NI, n=6) and STm-infected only (n=9) were included as controls. (A) Total number per spleen of red pulp macrophages (F4/80$^+$CD11b$^-$) and Ly6C$^+$ monocytes (F4/80$^+$CD11b$^+$Ly6G-Ly6C$^+$). Data combined from two independent experiments. Each point represents one mouse. Horizontal lines depict the median. Error bars depict the 75th-25th interquartile range (IQR). Statistical analyses were performed using the Kruskal-Wallis with Dunn's multiple comparisons test. (B) Spleen sections were stained for IgD (B cells, pink), CD169 (Marginal Zone Macrophages, blue), and SIGN-R1 (Metallophilic macrophages, red). WP=white pulp, RP=red pulp. (C) Representative immunohistochemistry (from 2 experiments, 4 mice per group) of livers obtained from C57BL/6 mice treated with clodronate liposomes before i.p. infection with 5x10^5 CFU STm SL3261. Mice were culled at day 7. Liver sections were stained for Clec4F (brown) by IHC. Red arrows show positive staining for Clec4F.

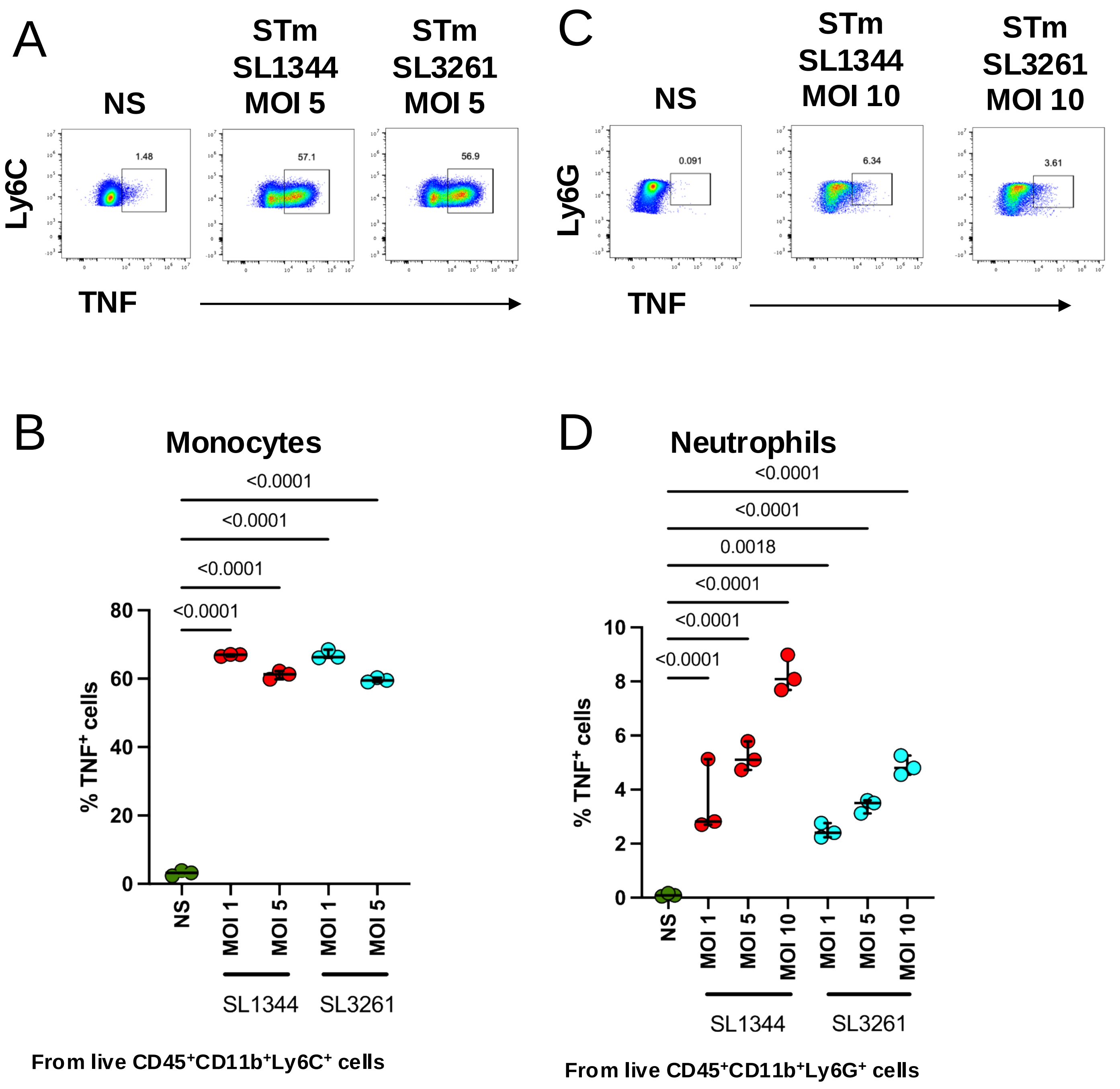

Supplementary figure 9. TNF can be induced in Ly6C+ monocytes and neutrophils in response to infection with STm in vitro. (A) Representative flow cytometry plots of intracellular TNF in live $CD45^+CD11b^+Ly6C^+$ cells. Monocytes were enriched from bone marrows from C57BL/6 by magnetic cell separation. Enriched cells were stimulated for 2 hours with STm SL3261 or SL1344 at a MOI of 1 or 5. (B) Frequency of TNF^+ monocytes. Each dot represents a replicate. (C) Representative FACS plots of intracellular TNF from live $CD45^+CD11b^+Ly6G^+$ cells. Neutrophils were enriched from bone marrows from C57BL/6 by Percoll gradient. Enriched neutrophils were stimulated for 2 hours with STm SL3261 or SL1344 at MOIs of 1, 5 and 10. (D) Frequency of TNF^+ neutrophils. Each dot represents a replicate. Horizontal lines show the median. Error bars depict the 75th-25th interquartile range (IQR). Statistical analyses were performed using the One-Way ANOVA with Dunnett's multiple comparisons test.

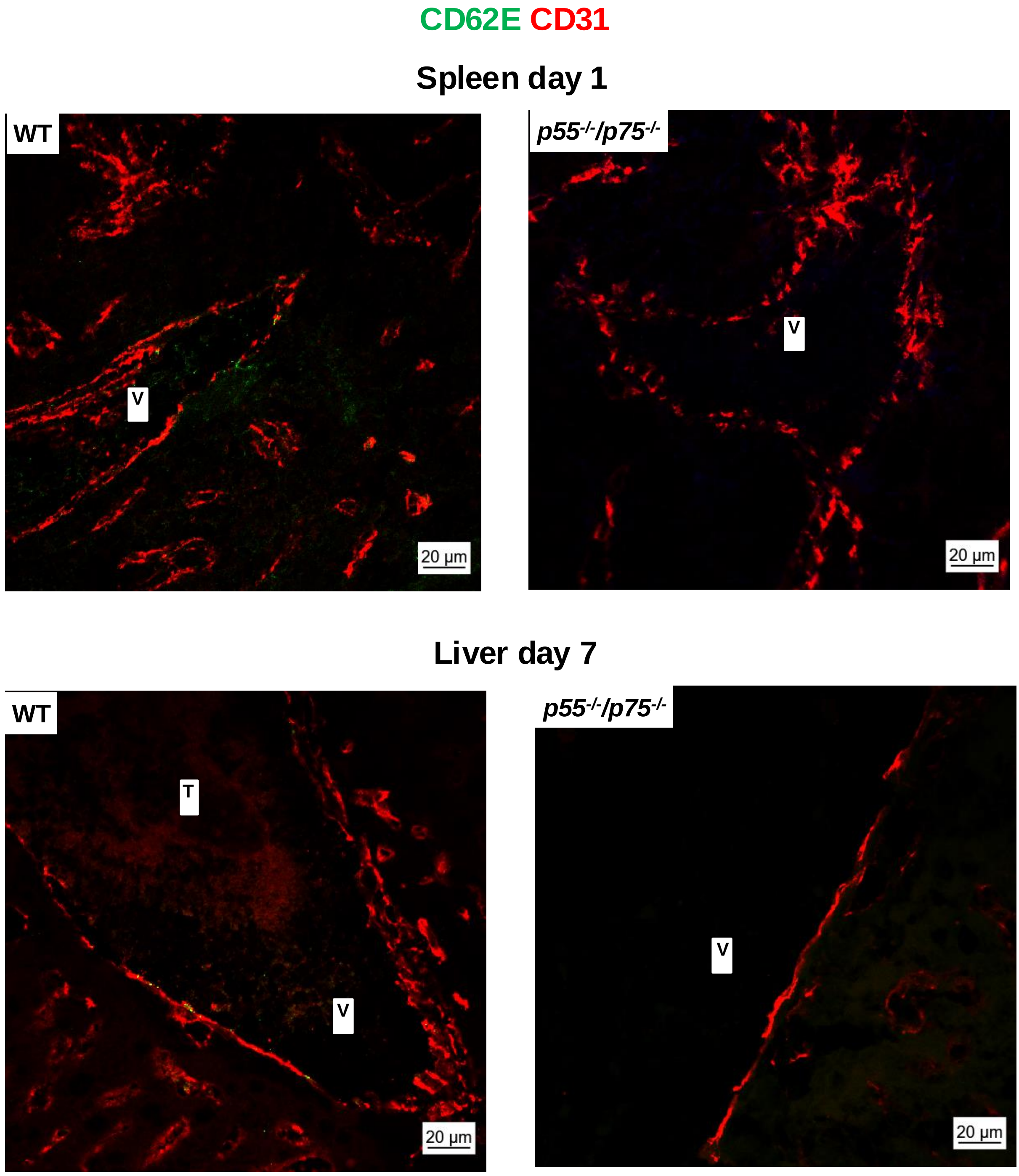

Supplementary figure 10. **TNF receptor-deficient mice do not induce CD62E in the spleen or liver after STm infection.** Representative images of spleen and liver sections from mice deficient in the TNF receptors subunits p55 and p75 (*p55*$^{-/-}$/*p75*$^{-/-}$) and WT controls, infected with STm for 1 day (spleen, upper row) or 7 days (liver, bottom row) described for. Sections were stained for CD62E (green) and CD31 (red). T=thrombus, V=vessel.

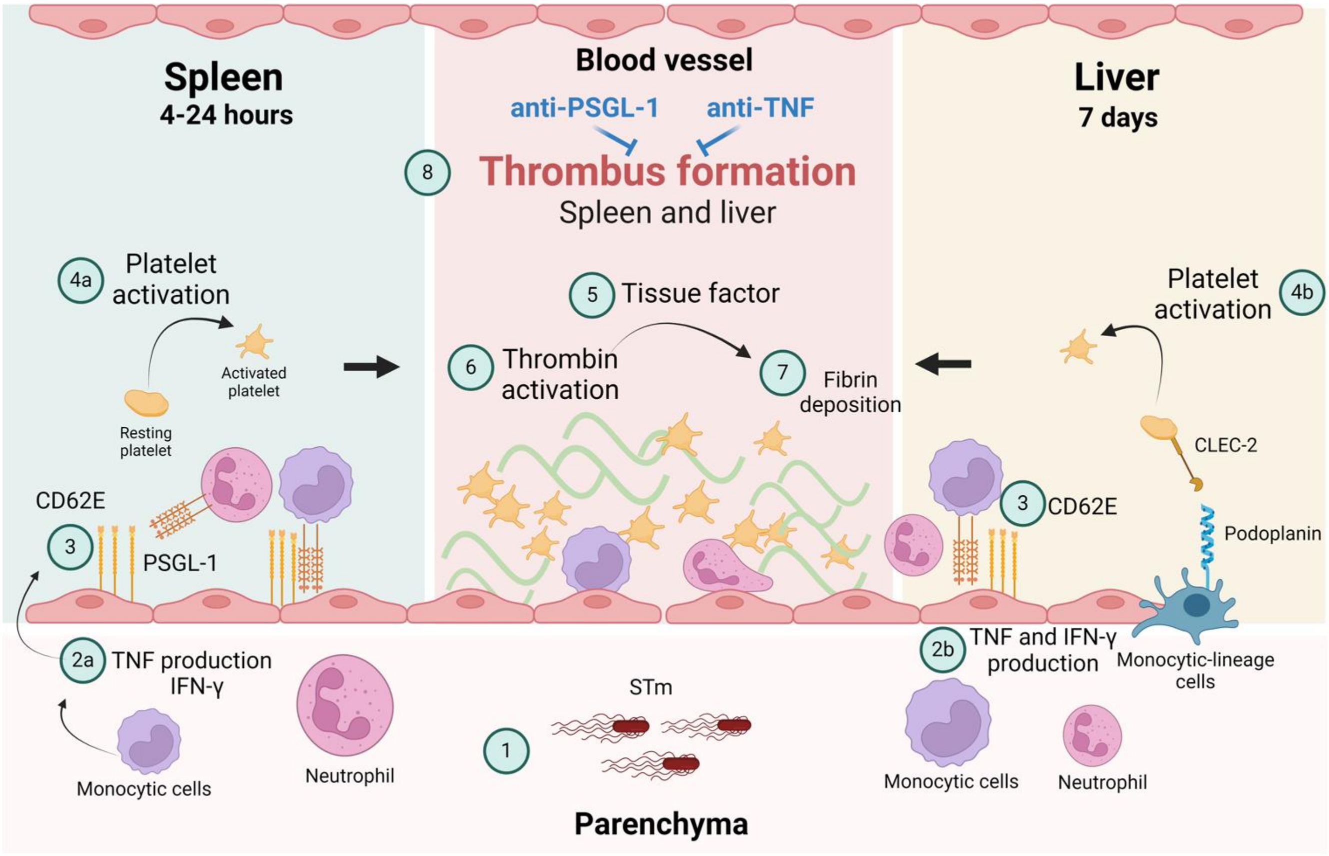

Supplementary figure 11. Proposed model for the induction of thrombosis by STm in the spleen and liver.

Spleen: STm invades the tissues including peri-vascular sites (1). This results in the local production of TNF and IFN-γ (2) and the further recruitment of phagocytes to parenchymal sites and proximal vessels. PSGL-1 and the upregulation of CD62E on the vasculature mediate neutrophil recruitment to the spleen and phagocyte interactions in the vasculature (3). Activation of platelets in the spleen occurs through an unknown mechanism (4a). The combination of TF expression on the subendothelium (5) and the activation of thrombin (6), promotes fibrin deposition (7) and results in thrombus development (8).

Liver: The pathway to thrombosis in the liver is largely conserved except for the additional critical need for IFN-γ (2b), which enhances local monocytic-lineage cell recruitment and the requirement for platelet expression of CLEC-2 (4b) which is required for platelet activation and thrombus formation (8). Finally, this process can be prevented in both organs by the administration of anti-PSGL-1 antibodies or anti-TNF. Created in BioRender. Perez toledo, M. (2025) https://BioRender.com/a52k104.

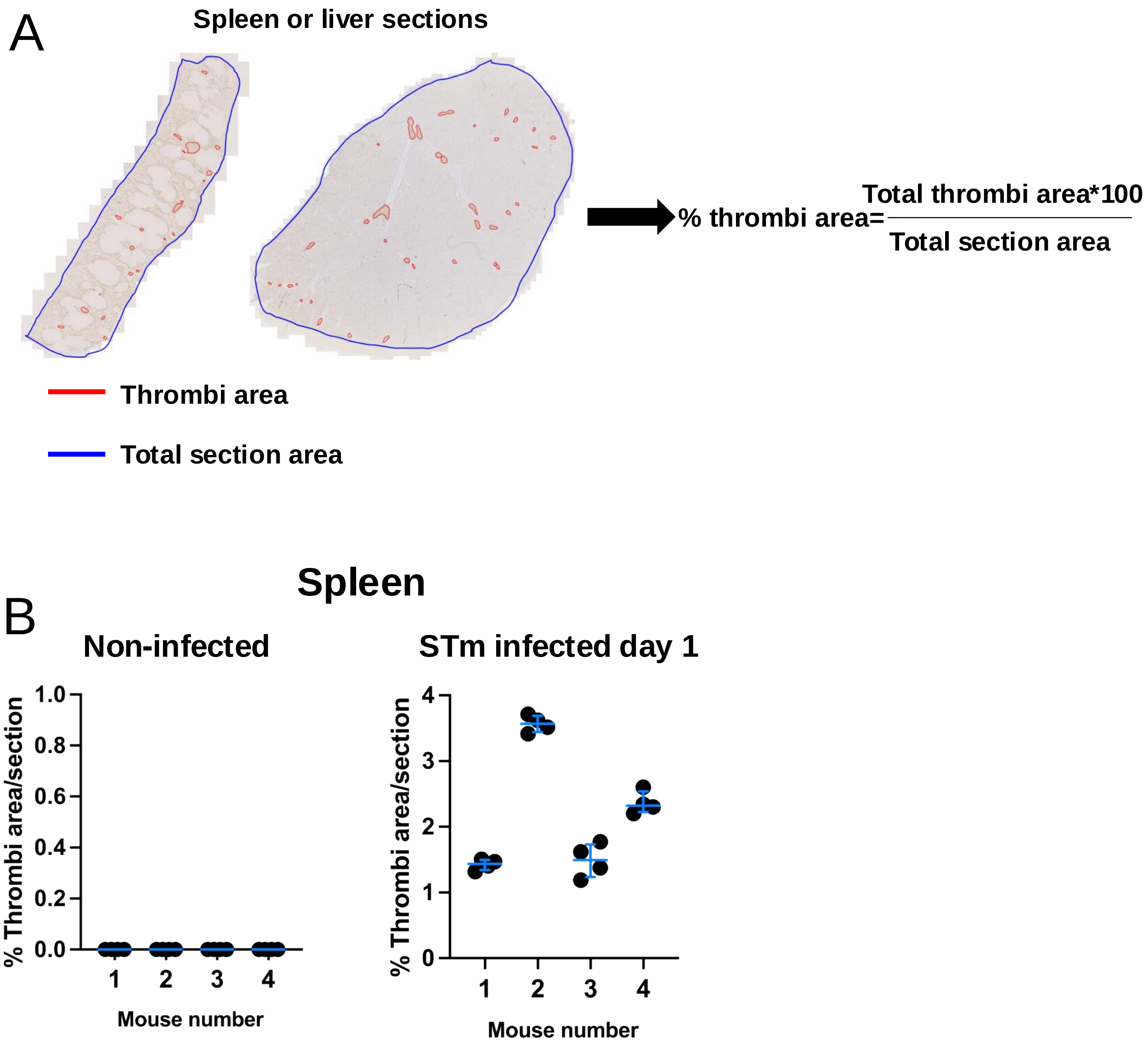

Supplementary figure 12. **Methodological approach for the quantification of thrombosis in organs.** (A) The diagram describes the process for quantification of thrombi in spleen and liver sections. Whole spleen and liver sections were stained for CD41 and fibrin by immunohistochemistry. Slides were scanned with a Zeiss Axio Scan Z1 and the analysis performed with Zen Blue v3.1. The total thrombi area was added, divided by the total section area, and multiplied by 100. (B) Serial spleen sections from non-infected (left) or STm infected for 1 day (right) were stained for CD41 and fibrin by immunohistochemistry, and the thrombi area was quantified per mouse. Each point represents a different section. Horizontal bars depict the median. Error bars depict the 75th-25th interquartile range (IQR).

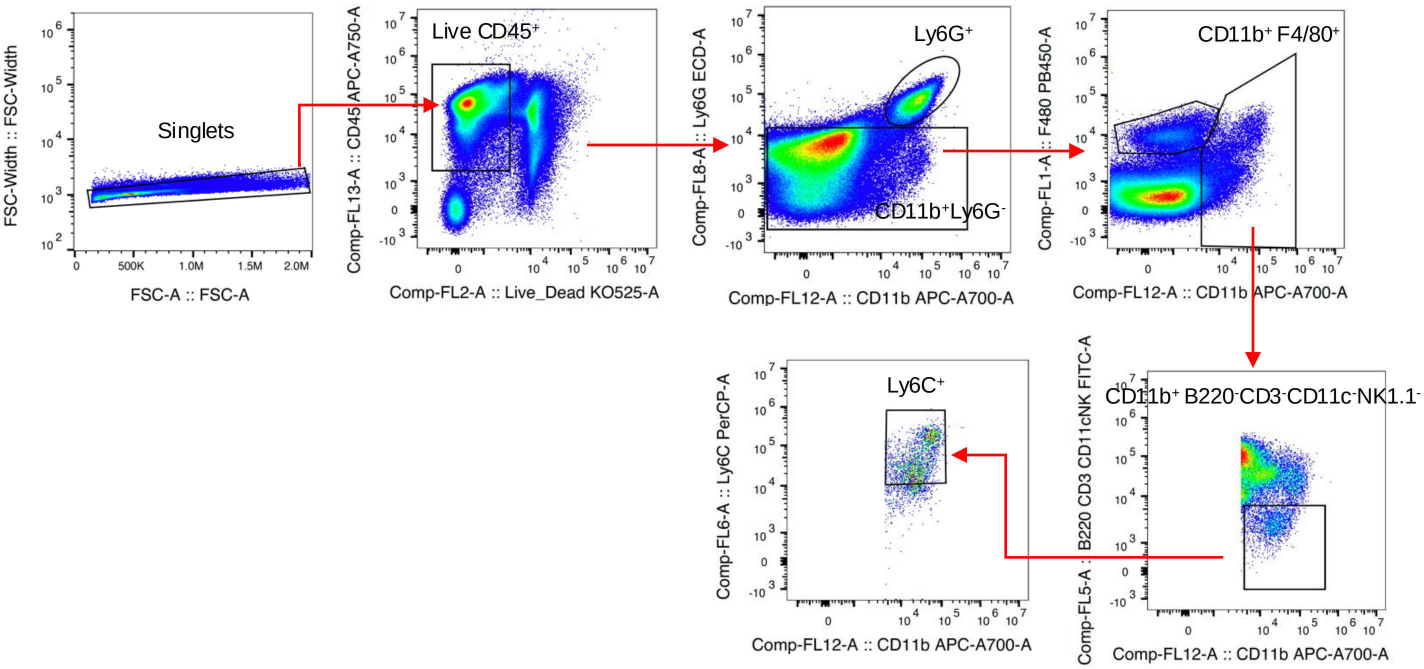

Supplementary figure 13. Representative FACS plots showing the gating analysis for neutrophils (CD11b+Ly6G+) cells and Ly6C+ monocytes (CD11b+Ly6G-B220-CD3-CD11c-NK1.1-Ly6C+).

Supplementary table I. Reagents used in this study

Target	Format	Manufacturer	Host	Clone	Cat. No.	Dilution
**Primary**						
CD41	Purified	eBioscience	Rat	eBioMWReg30	14-0411-85	1:300
Fibrin	Purified	Accurate chemical & Scientific corporation	Goat	Polyclonal		1:2000
Ly6G	Purified	BD Pharmingen	Rat	1A8	551459	1:600
Ly6G	Biotin	Biolegend	Rat	1A8	127604	1:600
F4/80	Alexa Fluor 488	Invitrogen	Rat	BM8	14-4801-82	1:300
F4/80	Alexa Fluor 488	Biolegend	Rat	BM8	123106	1:300
Ly6C	Biotin	Biolegend	Rat	HK1.4	128004	1:300
TNF	Purified	Abcam	Rabbit	Polyclonal	ab9739	1:200
CD31	Alexa Fluor 488	Invitrogen	Rat	390	14-0311-82	1:100
CD31	Biotin	Invitrogen	Rat	390	13-0311-83	1:100
CD62E	Purified	BD Pharmingen	Rat	10E9.6	550290	1:10
aSMA	Cy3	Sigma	Mouse	1A4	C6198	1:100
TF	Purified	R&D systems	Goat	polyclonal	AF3178	1:100
CD11b	Alexa Fluor 488	eBioscience	Rat	M1/70	17-0112-81	1:300
PSGL-1	Purified	BD Pharmingen	Rat	2PH1	564310	
Salmonella	Purified	Abcam	Rabbit	Polyclonal	ab35156	1:500
Citrullinated histone 3	Purified	Abcam	Rabbit	Polyclonal	ab5103	1:500
**Secondary**						
anti-rat	Biotin	Dako	Rabbit	Polyclonal	E0468	1:600
Anti-sheep	HRP	Jackson Immunoresearch	Donkey	Polyclonal	713035147	1:500
anti-rabbit	Alexa Fluor 488	Jackson Immunoresearch	Donkey	Polyclonal	711-545-152	1:500
anti-AF488	Alexa Fluor 488	Invitrogen	Rabbit	Polyclonal	710369	1:300
anti-rat	Alexa Fluor 647	Jackson Immunoresearch	Donkey	Polyclonal	712-605-153	1:500
Anti-rat	Cy3	Jackson Immunoresearch	Donkey	Polyclonal	712-165-153	1:500
Anti-sheep	Alexa Fluor 488	Jackson Immunoresearch	Donkey	Polyclonal	713-545-147	1:500
Vectastain® ABC-Alkaline Phosphatase	Alkaline Phosphatase	Vector Laboratories			AK-5000	
AF555 Streptavidin	Alexa Fluor 555	Invitrogen			S32355	1:1000
**Intravital microscopy**						
CD49b	PE	Biolegend	Arm Hms	HMα2	103506	1.6 µg/mouse
Ly6G	Brilliant Violent 421	Biolegend	Rat	1A8	127628	1.6 µg/mouse
F4/80	Alexa Fluor 647	Biolegend	Rat	BM8	123122	1.6 µg/mouse
Thrombin Activity Assay		Anaspec			AS-72129	8 µL of probe/mouse
**Flow Cytometry**						
Zombie Aqua Viability dye	Brilliant Violent 510	Biolegend			423101	1:1000
CD45	PE-Cy7		Rat	30-F11	103114	1:1000
CD11b	Alexa Fluor 700	BD Pharmingen	Rat	M1/70	557960	1:150
Ly6G	PE CF594	BD Horizon	Rat	1A8	562700	1:400
Ly6G	APC	BD Pharmingen	Rat	1A8	560599	1:300
Ly6C	PerCP Cy5.5	eBioscience	Rat	HK1.4	25-5932-82	1:300
F480	Brilliant Violent 421	Biolegend	Rat	BM8	123137	1:200
CD3	Alexa Fluor 488	eBioscience	Rat	145-2C11	53-0031-82	1:100
B220	FITC	BD Pharmingen	Rat	RA3-6B2	553087	1:100
NK1.1	FITC	BD Pharmingen	Rat	PK136	553164	1:50
TNF	PE Dazzle 594	Biolegend	Rat	MP6-XT22	506346	1:100
Cytofix/Cytoperm Fixation/permeabilization kit				-	554714	
**In vivo blocking/depleting**						
Invivo Mab anti-mouse Ly6G	Purified	BioXcell	Rat		BE0075-1	500 µg/ mouse
Clodronate Liposomes		Liposoma BV			C-005	1 mg/mouse
TNF	Purified	BioXcell	Rat	XT3.11	BE0058	500 µg/ mouse
Invivo Mab anti-mouse PSGL-1 (CD162)	Purified	BioXcell	Rat	4RA10		300 µg/ mouse
Rat IgG	Purified	Sigma	Rat	Polyclonal	I4131	500 µg or 300 µg/ mouse
