# Supplementary material for: Discrete and conserved inflammatory signatures drive thrombosis in different organs after Salmonella infection
Source: Nat Commun. 2025 Mar 10;16:2356. doi: 10.1038/s41467-025-57466-6 (PMC11894133; doi:10.1038/s41467-025-57466-6)
Supplement: Supplementary file 2 — Description of Additional Supplementary Files [file 41467_2025_57466_MOESM2_ESM.pdf]

Description of Additional Supplementary Files

Supplementary Movies

Supplementary Movie 1. Representative video of the spleen of a non-infected mouse. Mice were prepped for intravital microscopy as described in the methods section. Time lapse video with time shown in minutes, seconds and milliseconds. Red=CD49b, blue=Ly6G, grey=F4/80.

Supplementary Movie 2. C57Bl/6 mice were infected i.p. with $5x10^5$ CFU STm SL3261. Twenty-four hours later, mice were prepped for intravital imaging as described in the methods section. The video shows a representative field of view of a blood vessel in the spleen. Time lapse video with time shown in minutes, seconds and milliseconds. Red=CD49b, blue=Ly6G, grey=F4/80.

Supplementary Movie 3. C57Bl/6 mice were infected i.p. with $5x10^5$ CFU STm SL3261. 24 hours later, the mice were prepped for intravital imaging as described in the methods section. The video shows a representative field of view of a blood vessel in the spleen. The circles depict areas of interaction between platelets, neutrophils and monocytic cells. Time lapse video with time shown in minutes, seconds and milliseconds. Red=CD49b, blue=Ly6G, grey=F4/80.

Supplementary Movie 4. C57Bl/6 mice were infected i.p. with $5x10^5$ CFU STm SL3261. 24 hours later, the mice were prepped for intravital imaging as described in the methods section. The video shows a different field of view of a blood vessel in the spleen. Time lapse video with time shown in minutes, seconds and milliseconds. Red=CD49b, blue=Ly6G, grey=F4/80.

Supplementary Movie 5. Representative video of the spleen of a non-infected mouse. Mice were prepped for intravital microscopy as described in the methods section. Time lapse video with time shown in minutes, seconds and milliseconds. Red=GPIb, blue=Ly6G, green=F4/80.

Supplementary Movie 6. C57Bl/6 mice were infected i.p. with $5x10^5$ CFU STm SL3261. 6 hours later, mice were prepped for intravital imaging as described in the methods section. The video shows a different field of view of a blood vessel in the spleen. Time lapse video with time shown in minutes, seconds and milliseconds. Red=GPIb, blue=Ly6G, green=F4/80.

Supplementary Movie 7. Representative video of the liver of a non-infected mouse. Time lapse video with time shown in minutes, seconds and milliseconds. Red=CD49b, blue=Ly6G, grey=F4/80.

Supplementary Movie 8. C57Bl/6 mice were infected i.p. with $5x10^5$ CFU STm SL3261. 24 hours later, the mice were prepped for intravital imaging as described in the methods section. The video shows a representative field of view of the liver. Time lapse video with time shown in minutes, seconds and milliseconds. Red=CD49b, blue=Ly6G, grey=F4/80.
