# Supplementary material for: Discrete and conserved inflammatory signatures drive thrombosis in different organs after Salmonella infection
Source: Nat Commun. 2025 Mar 10;16:2356. doi: 10.1038/s41467-025-57466-6 (PMC11894133; doi:10.1038/s41467-025-57466-6)
Supplement: Supplementary file 11 — Reporting Summary [file 41467_2025_57466_MOESM11_ESM.pdf]

nature portfolio

Corresponding author(s): Adam F. Cunningham

Last updated by author(s): Dec 23, 2024

# Reporting Summary

Nature Portfolio wishes to improve the reproducibility of the work that we publish. This form provides structure for consistency and transparency in reporting. For further information on Nature Portfolio policies, see our Editorial Policies and the Editorial Policy Checklist.

## Statistics

For all statistical analyses, confirm that the following items are present in the figure legend, table legend, main text, or Methods section.

| n/a | Confirmed | |
|---|---|---|
| ☐ | ☒ | The exact sample size (*n*) for each experimental group/condition, given as a discrete number and unit of measurement |
| ☐ | ☒ | A statement on whether measurements were taken from distinct samples or whether the same sample was measured repeatedly |
| ☐ | ☒ | The statistical test(s) used AND whether they are one- or two-sided<br>*Only common tests should be described solely by name; describe more complex techniques in the Methods section.* |
| ☐ | ☒ | A description of all covariates tested |
| ☐ | ☒ | A description of any assumptions or corrections, such as tests of normality and adjustment for multiple comparisons |
| ☐ | ☒ | A full description of the statistical parameters including central tendency (e.g. means) or other basic estimates (e.g. regression coefficient) AND variation (e.g. standard deviation) or associated estimates of uncertainty (e.g. confidence intervals) |
| ☐ | ☒ | For null hypothesis testing, the test statistic (e.g. *F*, *t*, *r*) with confidence intervals, effect sizes, degrees of freedom and *P* value noted<br>*Give P values as exact values whenever suitable.* |
| ☒ | ☐ | For Bayesian analysis, information on the choice of priors and Markov chain Monte Carlo settings |
| ☒ | ☐ | For hierarchical and complex designs, identification of the appropriate level for tests and full reporting of outcomes |
| ☒ | ☐ | Estimates of effect sizes (e.g. Cohen's *d*, Pearson's *r*), indicating how they were calculated |

*Our web collection on statistics for biologists contains articles on many of the points above.*

## Software and code

Policy information about availability of computer code

| | |
|---|---|
| Data collection | No code was used to analyse data in this study |
| Data analysis | Sofware used for data analysis: Fiji (version 2.9.0), FlowJo Software v10.10, GraphPad Prism version 10.1.0 |

For manuscripts utilizing custom algorithms or software that are central to the research but not yet described in published literature, software must be made available to editors and reviewers. We strongly encourage code deposition in a community repository (e.g. GitHub). See the Nature Portfolio guidelines for submitting code & software for further information.

## Data

Policy information about availability of data

All manuscripts must include a data availability statement. This statement should provide the following information, where applicable:

- Accession codes, unique identifiers, or web links for publicly available datasets
- A description of any restrictions on data availability
- For clinical datasets or third party data, please ensure that the statement adheres to our policy

Source data are provided with this paper

# Research involving human participants, their data, or biological material

Policy information about studies with human participants or human data. See also policy information about sex, gender (identity/presentation), and sexual orientation and race, ethnicity and racism.

| | |
|---|---|
| Reporting on sex and gender | No human participants were used in this study |
| Reporting on race, ethnicity, or other socially relevant groupings | No human participants were used in this study |
| Population characteristics | No human participants were used in this study |
| Recruitment | No human participants were used in this study |
| Ethics oversight | No human participants were used in this study |

Note that full information on the approval of the study protocol must also be provided in the manuscript.

# Field-specific reporting

Please select the one below that is the best fit for your research. If you are not sure, read the appropriate sections before making your selection.

☒ Life sciences ☐ Behavioural & social sciences ☐ Ecological, evolutionary & environmental sciences

For a reference copy of the document with all sections, see nature.com/documents/nr-reporting-summary-flat.pdf

# Life sciences study design

All studies must disclose on these points even when the disclosure is negative.

| | |
|---|---|
| Sample size | Group sizes were determined using G*Power3.1.9.2 based on the expected degree of difference estimated between groups (informed partly by previous experimental studies or by pilot studies) to generate a study with a power of at least 80% and where p<0.05. Number of mice per group were assigned depending on the litter size availability and groups paired to similar age as much as possible. |
| Data exclusions | No data were excluded from this study |
| Replication | Experiments were repeated at least twice . All replication attempts were successful. |
| Randomization | No randomization was made when genetically-altered mice were used and were used based on availability and matching with WT mice. WT mice randomization was performed by animal technicians independent of the group randomly sourcing purchased mice to cages. |
| Blinding | Analyses were performed blindly by one member of the team and specified where appropriate in the methods section. Investigators were blinded during data analysis. |

# Reporting for specific materials, systems and methods

We require information from authors about some types of materials, experimental systems and methods used in many studies. Here, indicate whether each material, system or method listed is relevant to your study. If you are not sure if a list item applies to your research, read the appropriate section before selecting a response.

## Materials & experimental systems

| n/a | Involved in the study | |
|---|---|---|
| ☐ | ☒ | Antibodies |
| ☒ | ☐ | Eukaryotic cell lines |
| ☒ | ☐ | Palaeontology and archaeology |
| ☐ | ☒ | Animals and other organisms |
| ☒ | ☐ | Clinical data |
| ☒ | ☐ | Dual use research of concern |
| ☒ | ☐ | Plants |

## Methods

| n/a | Involved in the study | |
|---|---|---|
| ☒ | ☐ | ChIP-seq |
| ☐ | ☒ | Flow cytometry |
| ☒ | ☐ | MRI-based neuroimaging |

## Antibodies

| | |
|---|---|
| Antibodies used | CD41 Purified eBioscience Rat eBioMWReg30 14-0411-85 1:300 |

Antibodies used

Fibrin Purified Accurate chemical & Scientific corporation Goat Polyclonal 1:2000
Ly6G Purified BD Pharmingen Rat 1A8 551459 1:600
Ly6G Biotin Biolegend Rat 1A8 127604 1:600
F4/80 Alexa Fluor 488 Invitrogen Rat BM8 14-4801-82 1:300
F4/80 Alexa Fluor 488 Biolegend Rat BM8 123106 1:300
Ly6C Biotin Biolegend Rat HK1.4 128004 1:300
TNF Purified Abcam Rabbit Polyclonal ab9739 1:200
CD31 Alexa Fluor 488 Invitrogen Rat 390 14-0311-82 1:100
CD31 Biotin Invitrogen Rat 390 13-0311-83 1:100
CD62E Purified BD Pharmingen Rat 10E9.6 550290 1:10
aSMA Cy3 Sigma Mouse 1A4 C6198 1:100
TF Purified R&D systems Goat polyclonal AF3178 1:100
CD11b Alexa Fluor 488 eBioscience Rat M1/70 17-0112-81 1:300
PSGL-1 Purified BD Pharmingen Rat 2PH1 564310
Salmonella Purified Abcam Rabbit Polyclonal ab35156 1:500
Citrullinated histone 3 Purified Abcam Rabbit Polyclonal ab5103 1:500
anti-rat Biotin Dako Rabbit Polyclonal E0468 1:600
Anti-sheep HRP Jackson Immunoresearch Donkey Polyclonal 713035147 1:500
anti-rabbit Alexa Fluor 488 Jackson Immunoresearch Donkey Polyclonal 711-545-152 1:500
anti-AF488 Alexa Fluor 488 Invitrogen Rabbit Polyclonal 710369 1:300
anti-rat Alexa Fluor 647 Jackson Immunoresearch Donkey Polyclonal 712-605-153 1:500
Anti-rat Cy3 Jackson Immunoresearch Donkey Polyclonal 712-165-153 1:500
Anti-sheep Alexa Fluor 488 Jackson Immunoresearch Donkey Polyclonal 713-545-147 1:500
CD49b PE Biolegend Arm Hms HMα2 103506 1.6 µg/mouse
Ly6G Brilliant Violent 421 Biolegend Rat 1A8 127628 1.6 µg/mouse
F4/80 Alexa Fluor 647 Biolegend Rat BM8 123122 1.6 µg/mouse
CD45 PE-Cy7 Rat 30-F11 103114 1:1000
CD11b Alexa Fluor 700 BD Pharmingen Rat M1/70 557960 1:150
Ly6G PE CF594 BD Horizon Rat 1A8 562700 1:400
Ly6G APC BD Pharmingen Rat 1A8 560599 1:300
Ly6C PerCP Cy5.5 eBioscience Rat HK1.4 25-5932-82 1:300
F480 Brilliant Violent 421 Biolegend Rat BM8 123137 1:200
CD3 Alexa Fluor 488 eBioscience Rat 145-2C11 53-0031-82 1:100
B220 FITC BD Pharmingen Rat RA3-6B2 553087 1:100
NK1.1 FITC BD Pharmingen Rat PK136 553164 1:50
TNF PE Dazzle 594 Biolegend Rat MP6-XT22 506346 1:100
Invivo Mab anti-mouse Ly6G Purified BioXcell Rat BE0075-1 500 µg/ mouse
Clodronate Liposomes Liposoma BV C-005 1 mg/mouse
TNF Purified BioXcell Rat XT3.11 BE0058 500 µg/ mouse
Invivo Mab anti-mouse PSGL-1 (CD162) Purified BioXcell Rat 4RA10 300 µg/ mouse
Rat IgG Purified Sigma Rat Polyclonal I4131 500 µg or 300 µg/ mouse

Validation

All antibodies used in our study with the manufacturer and catalogue number are reported in supplementary table I

## Animals and other research organisms

Policy information about studies involving animals; ARRIVE guidelines recommended for reporting animal research, and Sex and Gender in Research

Laboratory animals

C57Bl/6J mice aged 6-8 weeks old were used in this study (strain code 632). Tissue Factor mice, IFN-gamma-deficient mice, PF4CreCLEC-2fl/fl mice and TNF receptor-deficient mice (p55-/-p75-/-), all in C57BL/6 background were used in this study.

Wild animals

No wild animals were used in this study

Reporting on sex

Male and female mice were used in this study

Field-collected samples

No field-collected samples were used in this study

Ethics oversight

Mice were used in accordance with the Home Office guidelines at the Biomedical Services Unit of the University of Birmingham under the project License P0677946

Note that full information on the approval of the study protocol must also be provided in the manuscript.

## Plants

| | |
|---|---|
| Seed stocks | No seed stocks were used in this study |
| Novel plant genotypes | No novel plant genotypes were created in this study |
| Authentication | No seed stocks or novel plant genotypes were created in this study |

## Flow Cytometry

### Plots

Confirm that:

☒ The axis labels state the marker and fluorochrome used (e.g. CD4-FITC).

☒ The axis scales are clearly visible. Include numbers along axes only for bottom left plot of group (a 'group' is an analysis of identical markers).

☒ All plots are contour plots with outliers or pseudocolor plots.

☒ A numerical value for number of cells or percentage (with statistics) is provided.

### Methodology

| | |
|---|---|
| Sample preparation | Single-cell suspensions from spleens were prepared by mashing approximately 20 mg of tissue through a 70 m cell strainer (Falcon). Red blood cells were lysed with ACK Lysis buffer (Gibco) and cells resuspended in RPMI with 10% FBS. Liver single-cell suspensions were prepared as described previously (8). Cells were stained for viability with Zombie Aqua (Biolegend, UK) prior incubation with primary antibodies during 30 minutes at 4ºC. For intracellular staining of TNFα, 5 x 106 cells were seeded in 48-well plates, stimulated with 5 g/mL of heat-killed STm, and incubated at 37ºC with 5% CO2, in the presence of Golgi Stop (BD Biosciences). After overnight incubation, extracellular staining was performed as described above. Intracellular staining was performed at room temperature using the BD Cytofix/CytopermTM fixation/permeabilization kit (BD Biosciences) according to the manufacturer's instructions. Data acquisition was performed with a CytoFLEX using the CytEXPERT software (Beckman Coulter), and data was analyzed with FlowJo Software v10.5 (Tree Star). Neutrophils were defined as CD11b+Ly6G+, and monocytes were defined as CD11b+Ly6G-CD3-B220-NK1.1-Ly6C+. The proportion of these cells is expressed as frequency from live CD45+ leukocytes. A list of all reagents used can be found in supplementary table I. |
| Instrument | Beckman Coulter Cytoflex |
| Software | Collection of data was performed with CytEXPERT (Beckman Coulter) and analysis of the data was performed with Flowjo. |
| Cell population abundance | No sorting was performed in this study |
| Gating strategy | Gating analysis was performed by selecting single events by FSC-A FSC-width gating, then selecting total cells, live CD45+ cells. Neutrophils were defined as CD11b+Ly6G+, and monocytes were defined as CD11b+Ly6G-CD3-B220-NK1.1-Ly6C+. Positive and negative gates were determined using the appropriate FMOs for each marker. |

☒ Tick this box to confirm that a figure exemplifying the gating strategy is provided in the Supplementary Information.
